# EMPOWERing Patients With Diabetes Using Profiling and Targeted Feedbacks Delivered Through Smartphone App and Wearable (EMPOWER): Protocol for a Randomized Controlled Trial on Effectiveness and Implementation

**DOI:** 10.3389/fpubh.2022.805856

**Published:** 2022-02-25

**Authors:** Yu Heng Kwan, Sungwon Yoon, Chuen Seng Tan, Bee Choo Tai, Wee Boon Tan, Jie Kie Phang, Ngiap Chuan Tan, Cynthia Yan Ling Tan, Yan Ling Quah, David Koot, Hock Hai Teo, Lian Leng Low

**Affiliations:** ^1^Department of Pharmacy, National University of Singapore, Singapore, Singapore; ^2^Program in Health Services and Systems Research, Duke-NUS Medical School, Singapore, Singapore; ^3^SingHealth Internal Medicine Residency Programme, Singapore, Singapore; ^4^Centre for Population Health Research and Implementation, SingHealth Regional Health System, SingHealth, Singapore, Singapore; ^5^Saw Swee Hock School of Public Health, National University of Singapore, National University Health System, Singapore, Singapore; ^6^Yong Loo Lin School of Medicine National University of Singapore, National University Health System, Singapore, Singapore; ^7^Population Health and Integrated Care Office, Singapore General Hospital, Singapore, Singapore; ^8^SingHealth Polyclinics, Singapore, Singapore; ^9^SingHealth Duke-NUS Family Medicine Academic Clinical Program, Duke-NUS Medical School, Singapore, Singapore; ^10^School of Computing, National University of Singapore, Singapore, Singapore; ^11^SingHealth Community Hospital, Singapore, Singapore; ^12^Department of Family Medicine and Continuing Care, Singapore General Hospital, Singapore, Singapore

**Keywords:** randomized controlled trial, diabetes, smartphone application, protocol, nudge

## Abstract

**Introduction:**

Type 2 diabetes mellitus (T2DM) poses huge burden and cost on the healthcare system. Mobile health (mHealth) interventions that incorporate wearables may be able to improve diabetes self-management. The aim of this randomized controlled trial (RCT) is to investigate the clinical and cost-effectiveness of personalized educational and behavioral interventions delivered through an EMPOWER mobile application (app) among patients with T2DM.

**Methods:**

This is a parallel two-arm randomized controlled trial (RCT). Patients with T2DM recruited from primary care will be randomly allocated in a 1:1 ratio to either intervention or control group. The intervention group will receive personalized educational and behavioral interventions through the EMPOWER app in addition to their usual clinical care. The control group will receive the usual clinical care for their T2DM but will not have access to the EMPOWER app. Our primary outcome is patient activation score at 12 months. Secondary outcomes will include HbA1c, physical activity level and diet throughout 12 months; quality of life (QoL), medication adherence, direct healthcare cost and indirect healthcare cost at 6 and 12 months.

**Discussion:**

This RCT will provide valuable insights into the effectiveness and implementation of personalized educational and behavioral interventions delivered through mobile application in T2DM management. Findings from this study can help to achieve sustainable and cost-effective behavioral change in patients with T2DM, and this can be potentially scaled to other chronic diseases such as hypertension and dyslipidemia.

## Introduction

The prevalence of diabetes for all age-groups worldwide is expected to increase from 2.8% in 2000 to 4.4% in 2030 (1). It is estimated that 5.0 million deaths is attributed to diabetes globally, and the total global health expenditure due to diabetes is around 673 billion US dollars (2). Diabetes ranks eighth among the leading causes of disability and years of life lost (DALYs), and the DALYs from diabetes increased by more than 80% between 2000 and 2019 (3). The traditional healthcare facility-based consultation model of episodic contact in chronic disease management is ineffective to address lifestyle factors such as diet and exercise. It is well-established that lifestyle interventions have greater impact in managing chronic diseases such as diabetes than medication (4). Lifestyle interventions are also the first line of treatment in diabetes management (5).

Mobile health (mHealth), which refers to the use of mobile computing, wearable sensors, and communication devices for the provision of health services and information (6), has shown promising results in diabetes management. A Cochrane review indicates that computer-based diabetes self-management in patients with diabetes showed a beneficial effect on blood glucose control, and the effect was larger in the mobile phone subgroup (7). However, the effect of automated personalized educational and behavioral feedback has not been thoroughly investigated among patients with diabetes. Given the constraint in resources in primary care settings (8, 9), automated mHealth behavior change interventions may be more sustainable in diabetes management. Although previous study has investigated the effect of automated real-time educational, behavioral, and motivational messaging (10), the lack of appropriate behavior change theories in the study may have limited the effectiveness of the intervention. mHealth interventions are more likely to be effective if the selection and combination of behavior change techniques is informed by appropriate behavior change theories (7).

The aim of this randomized controlled trial (RCT) is to investigate the clinical and cost-effectiveness of the personalized educational and behavioral interventions delivered through mobile application among primary care patients with type 2 diabetes mellitus (T2DM). A successful execution of the RCT will provide insights on novel interventions to inculcate behavioral and lifestyle changes among patients with T2DM, which can reduce complications of T2DM and healthcare resources utilization through a cost-effective and sustainable approach.

## Methods and Analysis

**Figure 1:**
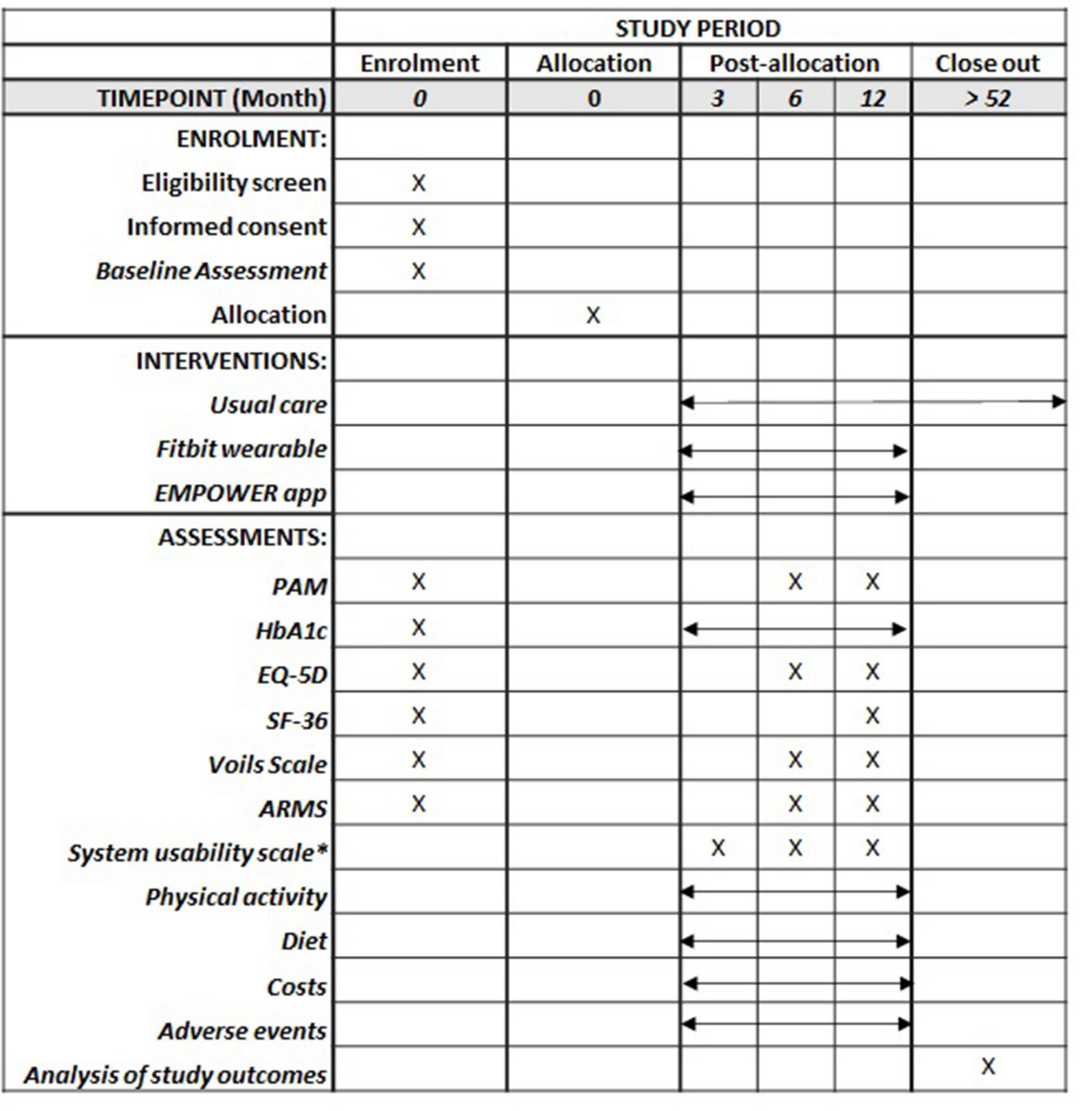
SPIRIT figure for the schedule of enrolment, interventions, and assessments. ARMS, adherence to refills and medication scale; EQ-5D-5L, EuroQoL-5 dimensions; HbA1c, glycated hemoglobin; PAM, patient activation measure; SF36-v2, Short Form-36 Version 2. *Only for participating randomized to intervention group.

This protocol is written in accordance with the Standard Protocol Items: Recommendations for Interventional Trials (SPIRIT) reporting guidelines (Figure 1) (11). This paper is based on protocol version 1.6 dated 10th September 2021.

### Study Design

**Figure 2:**
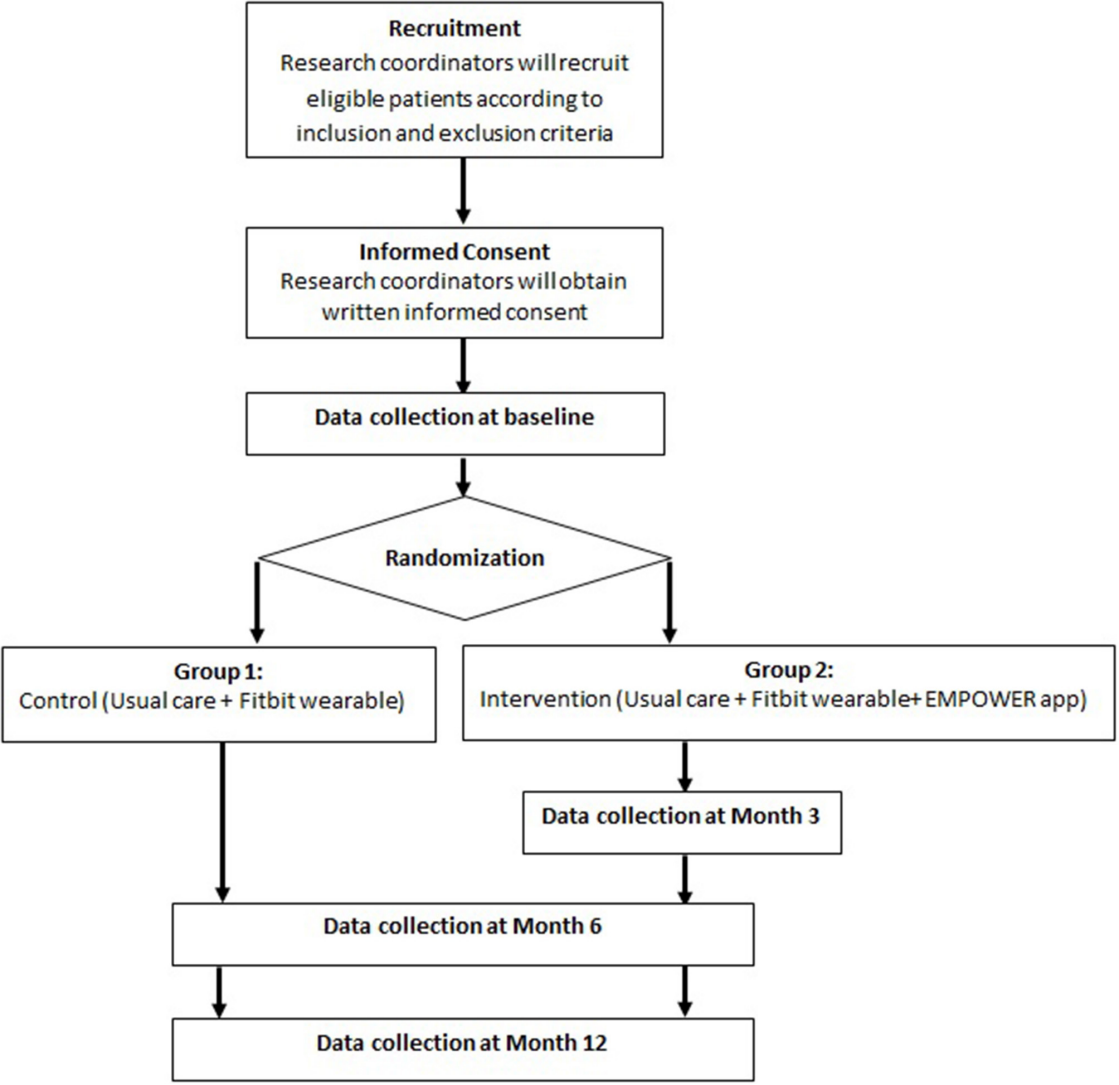
Trial work plan. Follow-up will be performed at month 3, 6, and 12 after baseline visit.

This study is a pragmatic parallel 2-arm randomized controlled trial (RCT) anchored on the Pragmatic Explanatory Continuum Indicator Summary Framework-2 (PRECIS-2) criteria (12). Patients with T2DM will be randomly allocated in a 1:1 ratio to either the intervention or control group. The intervention group will receive personalized educational and behavioral nudges delivered through the EMPOWER application (app) on top of their usual clinical care. The control group will receive the usual clinical care but will not have access to the EMPOWER app (Figure 2). This study has been approved by the SingHealth Centralized Institutional Review Board (Reference number: 2020/3093) and is registered on clinicalTrials.gov (NCT04518566).

### Eligibility Criteria

Patients will be recruited from three polyclinics (public healthcare centers which provide subsidized primary care). We will recruit participants who fulfill the inclusion criteria as follows: (1) Aged 40 years and above at time of recruitment (2) Have been diagnosed with diabetes at time of recruitment (3) Most recent glycated hemoglobin (HbA1c) ≥ 7.0% mmol/l (4) Physically able to exercise (5) Literate in English (6) Agreeable to be monitored by EMPOWER and/or Fitbit apps (7) Able to conform to monitoring schedule (Table S1). The exclusion criteria are patients who are (1) On insulin treatment (2) Require assistance with basic activities of daily living (BADL) (3) Have planned major operation or surgical procedure in the coming year at the time of recruitment (4) Cognitively impaired (scored < 6 on the Abbreviated Mental Test).

### Intervention

The participants in the intervention group will be given access to the EMPOWER app and Fitbit tracker. The EMPOWER app aims to nudge the participant toward successful self-management of their diabetes condition by encouraging and reinforcing positive lifestyle behaviors in three main aspects—diet, physical activity, medication adherence. The EMPOWER app is designed using the Behavior Change Wheel (BCW) as a guiding framework (13), and the following key functionalities of the EMPOWER app can be mapped to the persuasion, enablement, incentivization and education interventions in the BCW (Table 1).

**Table 1:** EMPOWER app features.

**App features**	**Description**	Intervention function^*^
Personalized notifications	Personalized notifications generated based on the user's inputs and predictive modeling	Enablement, education, persuasion
Gamification	Monetary reward for logging	Incentivisation
Educational resources	Instant access to diabetes management information	Education
Daily logs, report and diary	Monitor physical activity, diet, medication, weight, and blood glucose readings	Education
Goal setting	Set goals on physical activity, diet, medication, weight, and blood glucose	Enablement

* *Based on behavioral change wheel (14)*.

#### Personalized Nudges Generated Based on User's Inputs and Predictive Modeling

Notifications included content based on behavioral change technique including feedback on performance, positive reinforcement, and prompts to self-monitor (15). Each notification focused on a single behavior (physical activity, medication adherence or diet). The notifications will be tailored based on the risk profiles from predictive modeling.

#### Gamification

The bingo game encourages the user to input data logs (i.e., meal logs, medication logs) consistently. In the EMPOWER app, users gain points through the bingo game, and upon accumulation of sufficient points, they will be able to redeem prizes such as vouchers. In order to play the bingo game, users need to gain chances through the completion of daily logs - the more consistent they are in logging, the more chances they accumulate. The user is allowed to choose a specific word card, and can then complete each letter card. For example, if the user chooses the word FIT, they would have to complete three alphabet cards. Each chance allows the user to roll for a number once, and the number of points gained depends on the location of the specified number within the letter cards. If the number does not lie within the structure of the alphabet, the user gains less points. If the number lies within the structure of the alphabet, the user is one step closer to completing the alphabet card, and is rewarded a higher number of points. The aim is to get encourage and positively reinforce healthy lifestyle behavior.

#### Daily Logs, Report, and Diary

Participants can create diet, medication, blood glucose and weight log in the EMPOWER app. The physical activity and sleep data will be tracked by the Fitbit tracker.

#### Goal Setting

Participants can create goals for physical activity, blood glucose, diet, medication, and weight in the EMPOWER app.

#### Educational Resources

Educational resources on diabetes management verified by physiotherapists and dieticians are available in some of the notifications.

### Control

The participants in the control group will not have access to the EMPOWER app. However, they will receive Fitbit tracker, and will be instructed to download the Fitbit app.

### Outcome Measure

The primary outcome is the patient activation scores at 12 months (Table 2). The patient activation score will be measured using the 13-item patient activation measure (PAM) (16) which is developed with a focus on the skills, knowledge and beliefs required for patients to successfully manage chronic illnesses (17). The PAM has been validated in Singapore (18), and found to be highly correlated with several health indicators such as health behaviors and healthcare utilization (19, 20).

**Table 2:** Primary and secondary outcomes.

**Outcome**	**Source & instrument**	**Data collection timepoint**
**Main primary outcome**		
Patient activation	Survey—PAM	Baseline, 12 months
**Secondary outcomes**		
HbA1c	Electronic medical records	All valid test results throughout 12 months
Physical activity	Wearable—Number of steps, moderate to vigorous active minutes	Real-time tracking throughout 12 months
Diet	EMPOWER app—Calorie intake, carbohydrates and sugar intake	Real-time syncing of participant's self-entry of diet logs throughout 12 months
Medication adherence	Survey—Voils Scale, ARMS	Baseline, 6, 12 months
Quality of life	Survey—EQ-5D-5L, SF36-v2	• EQ-5D-5L: Baseline, 6, 12 months • SF36-v2: Baseline, 12 months
Direct healthcare cost	Electronic medical records–Cost of medical consultation, laboratory tests, medication, admissions and other healthcare utilization	All entries throughout 12 months
Indirect healthcare cost	Survey—Self-reported income, number of days of work missed due to medical condition, travel cost, employment status	Baseline, 6, 12 months

*ARMS, adherence to refills and medications scale; EQ-5D-5L, EuroQoL-5 dimensions; HbA1c, glycated hemoglobin; PAM, patient activation measure; SF36-v2, Short Form-36 Version 2*.

Secondary outcomes will include HbA1c, physical activity level and diet throughout 12 months; quality of life (QoL), medication adherence, direct healthcare cost and indirect healthcare cost at 6 and 12 months. QoL will be measured using the 36-Item Short Form Survey (SF-36) (21) and EQ-5D-5L (22, 23), with SF-36 only being assessed at baseline and 12 months while EQ-5D-5L being assessed at baseline, 6 and 12 months. Physical activity level will be measured by number of steps, and moderate to vigorous active minutes (MVPA). Diet will be assessed using calorie intake, carbohydrates and sugar intake derived from participant's self-entry of diet logs. Medication adherence will be measured using the self-report measure by Voils (24) and the Adherence to Refills and Medications Scale (ARMS) (25). The SF-36 (21, 26), EQ-5D-5L (23, 27), and self-report measure by Voils (28) have been validated in Singapore. Healthcare cost will be extracted from the electronic medical records, and supplemented with survey data regarding self-reported income, number of days of work missed due to medical condition, travel cost, employment status.

### Sample Size Calculation

We based our sample size calculation from a study by Solomon et al. (29). Although the commonly accepted minimal clinically important difference (MCID) for the PAM is 5 (30), there have been some studies suggesting that a MCID of <5 may be appropriate (31), and for this reason we have elected to use a conservative MCID of 2.5 in the sample size calculation. With a conservative estimate of 2.5 point difference on a 100-point scale in patient activation scores between the two arms with 1 pre- and 1 post-intervention (12 months) measurement, and assuming a standard deviation of 14 for both arms and correlation of 0.5, approximately 371 patients are needed for each arm to obtain a statistical power of 80% (two sided Type I error rate of 0.05) based on a 1:1 treatment allocation. After taking into account a dropout rate of approximately 20%, a total of 1,000 patients with 500 patients per arm will be needed for this trial.

### Randomization and Blinding

Participants will be allocated to the intervention group or control group using a randomization list pre-generated by a biostatistician. Stratified block randomization will be used with recruitment site as the stratification factor with 1:1 allocation ratio.

After a participant is enrolled in the study, a research coordinator will contact the personnel holding onto the randomization list (headquarter staffs who are not involved in patient recruitment) to reveal the participant's assigned group. The research coordinator will not be aware of the subsequent allocation prior to contacting the personnel holding onto the randomization list. Due to the nature of the intervention, it is not possible to blind study participants to group assignment.

### Statistical Analyses

All participants will be analyzed using intention-to-treat approach. Participant's characteristics will be summarized using mean (standard deviation) or median (interquartile range) as appropriate for continuous variables, and count and percentage for categorical variables. Primary outcome of patient activation score at 12 months will be analyzed using the analysis of covariance (ANCOVA), with adjustment made for baseline patient activation score and other confounding variables (e.g., participation in other ongoing health programmes).

For secondary outcomes with repeated measurements (e.g., quality of life, medication adherence, HbA1c), a linear mixed model will be used to account for within-individual correlation among measurements, and the sandwich estimator to obtain robust standard error estimates. The intervention indicator and time factor will be included as linear predictors adjusting for the respective baseline covariates. All evaluations will be made assuming a two-sided type I error rate of 0.05.

Economic evaluation will be conducted from both healthcare system and societal perspectives. Cost-effectiveness analysis (i.e., cost of reduction in 1 patient activation point) and cost-utility analysis (cost of reduction in 1 quality-adjusted life year saved) will be performed. For cost-effectiveness analysis, effectiveness will be measured as the improvement in patient activation scores at 12 months. For cost-utility analysis, utility will be measured using the EQ-5D-5L at all observed time points. The study team will regress the effectiveness on follow-up time in order to assess the time profile of the effectiveness of the intervention. In case of residual effectiveness at 12 months, analysis will extrapolate using the estimated effectiveness profile in order to assess additional effectiveness arising beyond study completion. Utility weights from Singapore will be applied to determine the corresponding societal preferences. All diabetes-related and non-diabetes-related health care use (secondary economic outcomes) will be used for the health care system perspective. Non-healthcare financial consequences (secondary economic outcomes) will be added for the societal perspective. All costs will be adjusted to 2017 values using consumer price index health care component. Sensitivity analysis will be conducted to evaluate the influence of uncertainties in the variables and assumptions employed on the analysis results.

### Implementation

Qualitative interviews with a purposive sample of patients and stakeholders (e.g., primary care physicians, healthcare professionals or management) will be conducted to collect their feedback on the EMPOWER app and nudges in educating and motivating behavior change for T2DM based on intervention components embedded to theoretical domain framework (32, 33). The qualitative data will be supplemented with data from the system usability scale (34), which will be completed by participants in the intervention group at month 3, 6, and 12. We will also conduct process evaluation on participant perceptions and contextual feasibility based on appropriate implementation science framework and/or theory.

### Data Management

All hardcopy source documents will be kept in locked cabinets at the individual recruitment sites. The locked cabinets can only be accessed by authorized personnel. All survey data will be entered into Research Electronic Data Capture (REDCap), a secure web application, by trained study staff via password-protected laptops. Electronic medical records data will only be available with the patient's consent, and the data will be stored in password-protected files prior to entry to REDCap. Fitbit data and EMPOWER app data will be stored on a password-protected secured cloud platform, and only the development team will have access to this platform. All data will be maintained for 7 years after study termination, at which point it will be disposed of in accordance to policy.

To ensure the quality of the information is consistent, there will be random audit of 3 cases against case report forms to check for entry errors on a monthly basis. For the data retrieved from 3rd party application programming interfaces (e.g., Fitbit), the development team will carry out random inspections periodically on the database records, as well as verify against records stored in the 3rd party databases. The principal investigator will oversee all data entry and proper data monitoring and audit procedures.

### Data Monitoring

An independent data and safety monitoring board will provide one interim monitoring of the un-blinded safety and effectiveness data for the trial after 500 participants have been recruited and completed 6 months follow-up. The data and safety monitoring board, comprising independent clinical experts and biostatistician, will help ensure the safety and proper conduct of the trial. Adverse events related to intervention throughout the study duration (12 months) will be recorded.

## Discussion

To our best knowledge, this study is the first to determine the clinical and cost-effectiveness of personalized educational and behavioral interventions including automated nudges through mobile application informed by appropriate behavior change theory (behavioral change wheel) (14) for T2DM management through a RCT study design. With the increasing manpower strain in healthcare settings (35, 36), automated personalized nudges present a unique opportunity to improve chronic disease self-management, as the use of fully-automated nudges may ensure scalability of the intervention in healthcare settings with limited manpower resources. Previous studies on mHealth interventions for diabetes self-management have predominantly focused on Western healthcare settings (37, 38), therefore this study which is conducted in an Asian healthcare settings may be useful for policymakers and researchers in similar socio-cultural contexts.

The study will also provide insights into the implementation in using mHealth and automated nudges in T2DM management through our cost-effectiveness analysis and qualitative interviews with relevant stakeholders, which will allow clinicians and researchers to gain insights into the design and implementation of the novel mobile app-based personalized educational and behavioral interventions including automated nudges. Ultimately, findings from this trial may help to achieve sustainable and cost-effective behavioral change in diabetes patients through patient empowerment and targeted chronic disease care.

## Ethics Statement

This study has been approved by the SingHealth Centralized Institutional Review Board (Reference number: 2020/3093). Written informed consent will be obtained from all participants. Protocol amendments, adverse effects reporting and annual review will be overseen by the SingHealth Centralized Institutional Review Board. We will disseminate the findings from this study through several ways, including presenting the study findings at international conferences relating to diabetes and/or digital health and publishing in peer-reviewed journals.

## Author Contributions

YK and SY conceived and designed the study and drafted the manuscript. BT and CST designed the statistical plan. WT participated in the design on the intervention and study. JP drafted the manuscript. NT, CYLT, YQ, and DK designed the study. HT designed the intervention. LL conceived and designed the study, drafted the manuscript, and solicited the funding. All authors read and approved the final manuscript.

## Funding

This work was supported by the Singapore Ministry of Health's National Innovation Challenge Grant (Ref: MOH/NIC/CDM1/2018) and Singapore Ministry of Health's National Medical Research Council under the SingHealth Regional Health System, Population-based, Unified, Learning System for Enhanced and Sustainable (PULSES) Health Centre Grant (NMRC/CG/C027/2017_SHS).

## Conflict of Interest

The authors declare that the research was conducted in the absence of any commercial or financial relationships that could be construed as a potential conflict of interest.

## Publisher's Note

All claims expressed in this article are solely those of the authors and do not necessarily represent those of their affiliated organizations, or those of the publisher, the editors and the reviewers. Any product that may be evaluated in this article, or claim that may be made by its manufacturer, is not guaranteed or endorsed by the publisher.
