# Supplementary material for: EMPOWERing Patients With Diabetes Using Profiling and Targeted Feedbacks Delivered Through Smartphone App and Wearable (EMPOWER): Protocol for a Randomized Controlled Trial on Effectiveness and Implementation
Source: Front Public Health. 2022 Feb 25;10:805856. doi: 10.3389/fpubh.2022.805856 (PMC8913889; doi:10.3389/fpubh.2022.805856)
Supplement: Supplementary file 1 [file Table_1.docx]

Supplementary table 1: Monitoring schedule

| **Activity** | **Minimum Requirements** |
| --- | --- |
| Wearing of Fitbit wearable | At least 8 hours a day, on at least 7 days a month (compromising at least 5 weekdays and 2 weekends) |
| Wearing of Fitbit to sleep | At least 1 weekday (Sunday - Thursdays night) and 1 weekend (Friday – Saturday nights) a week |
| Meal logging | All meals a day on at least 3 days (compromising 2 weekdays and 1 weekend) a week |
| Medication logging | All medications a day on at least 3 days (compromising 2  weekdays and 1 weekend) a week |
